# An Evaluation of Low-Cost Vision Processors for Efficient Star Identification

**DOI:** 10.3390/s20216250

**Published:** 2020-11-02

**Authors:** Surabhi Agarwal, Elena Hervas-Martin, Jonathan Byrne, Aubrey Dunne, Jose Luis Espinosa-Aranda, David Rijlaarsdam

**Affiliations:** 1 Intel Corporation, Intel R&D Ireland Ltd, Collinstown, Collinstown Industrial Park, Co., W23 CX68 Kildare, Ireland; jonathan.byrne@intel.com (J.B.); davidrijlaarsdam@outlook.com (D.R.); 2 Ubotica Technologies, DCU Alpha, Old Finglas Road, Glasnevin, D11KXN4 Dublin, Ireland; elena.hervas@ubotica.com (E.H.-M.); aubrey.dunne@ubotica.com (A.D); josel.espinosa@ubotica.com (J.L.E.-A.)

**Keywords:** power measurement, Myriad 2, STM32, OpenMV, star identification, star tracker, deep learning, star feature extraction

## Abstract

Star trackers are navigation sensors that are used for attitude determination of a satellite relative to certain stars. A star tracker is required to be accurate and also consume as little power as possible in order to be used in small satellites. While traditional approaches use lookup tables for identifying stars, the latest advances in star tracking use neural networks for automatic star identification. This manuscript evaluates two low-cost processors capable of running a star identification neural network, the Intel Movidius Myriad 2 Vision Processing Unit (VPU) and the STM32 Microcontroller. The intention of this manuscript is to compare the accuracy and power usage to evaluate the suitability of each device for use in a star tracker. The Myriad 2 VPU and the STM32 Microcontroller have been specifically chosen because of their performance on computer vision algorithms alongside being cost-effective and low power consuming devices. The experimental results showed that the Myriad 2 proved to be efficient and consumed around 1 Watt of power while maintaining 99.08% accuracy with an input including false stars. Comparatively the STM32 was able to deliver comparable accuracy (99.07%) and power measurement results. The proposed experimental setup is beneficial for small spacecraft missions that require low-cost and low power consuming star trackers.

## 1. Introduction

As advancements are being made in the spacecraft industry, there has been a massive growth in the number of low-cost satellite systems, such as CubeSats and SmallSats [1]. To launch small spacecraft missions successfully, lightweight attitude determination sensors that consume minimum amounts of power are required. There are several options including Sun sensors, Earth sensors, and horizon crossing indicators that are proficient in accurate attitude determination [2], but the best accuracy is generally achieved using star trackers [3]. Star trackers, also known as star sensors, are optical devices that determine the spacecraft’s attitude through imaging the sky, creating a star scene. A star identification algorithm then identifies the present stars by comparing the scene to a local database, the matched stars allow for the determination of attitude, solving the so-called lost-in-space problem.

Traditional star trackers are bulky and high power (greater than 5 Watts), and beyond the power and mass budget of small satellites [2]. For example, the power consumption of a commercially available star tracker is approximately 10 Watts and the mass is around 3 kg [4]. This is not suitable for a SmallSat. Accordingly, there is now an impetus to develop light-weight, low-cost star trackers that use Commercial-Off-The-Shelf (COTS) hardware suitable for vision systems and novel tracking approaches, such as the use of neural networks and deep learning, which provide constant search time rather than a time-consuming database lookup [5]. To this end, this work carries out a power measurement experiment running a Neural Network (NN) based star tracker on two low-cost and low power processors.

This work has the following outline:

- The two processors—the Myriad 2 VPU and the STM32 Microcontroller have been chosen to perform evaluations concerning the accuracy, frame rate, and power. This helps in understanding which processor will be best suited for the low-power consumption star tracker
- An efficient and robust NN-based star identification algorithm [6] has been used in this work to perform the testing with the two processors. This algorithm has high analytical performance and is robust to false stars.
- Through the above experiment, a processor can be selected based on the needs of the star tracker keeping in mind the accuracy, Frames Per Second (FPS), and power consumption.

## 2. Background

Recently, a new lost in space star identification algorithm similar to [7] has been proposed in [6] that performs the star tracking task using an NN Artificial Intelligence (AI) based approach. This approach is capable of achieving and exceeding the performance of traditional algorithmic-based approaches on synthetic datasets. However, the implementation of such NN-based star trackers has not previously been considered. Moreover, it is not only necessary to have star trackers with accurate attitude determination but also low-power consumption and low-cost to make sure SmallSat missions function effectively for long durations. As mentioned in Section 1, traditional star trackers are substantially large, power consuming, and out of the budget of SmallSats. To provide a cost-effective yet low-power and efficient solution to the star tracker approach, this manuscript examines two alternative low-power and low-cost hardware architectures for implementing the NN-based star tracker.

### 2.1. Hardware

The power measurement experiment carried out in this manuscript utilizes the Myriad 2 VPU from Intel Movidius and the STM32 Microcontroller from STMicroelectronics. These processors have been chosen to perform this experiment for a variety of reasons. Firstly, since these devices are Commercial-Off-The-Shelf (COTS) parts, they have a significantly lower price point than the radiation-hardened star tracker devices. This opens up the possibility of developing a low-cost star tracker for SmallSats. Furthermore, this low cost facilitates the use of multiple star trackers on the same SmallSat platform for redundancy. Secondly, the processor speed is several generations ahead of contemporary radiation-hardened silicon processes. Thirdly, as these devices are both targeted at embedded deployments in commercial and consumer applications where power is a priority, both devices have been designed to operate within extremely low power envelopes. Finally both devices are available as standalone products that enabled the evaluation of the processors without the requirement for custom designed hardware.

The Myriad 2 has undergone radiation characterization campaigns to assess its suitability for in-flight space deployment [8]. These campaigns, including both Total Ionising Dose (TID) and Single Event Effect (SEE) tests, have shown the Myriad 2 to be resilient to cumulative ionizing radiation up to 49 krad, and have demonstrated that the Myriad 2 does not have any critical latch-up sensitivities up to a Linear Energy Transfer (LET) of 8.8 MeVcm ${}^{2}$/mg. The Myriad 2 is already being deployed on a Low Earth Orbit (LEO) mission called Φ-sat-1 [9] and is scheduled for integration into two further missions for launch in 2022. In addition to its positive radiation characterization results and its flight heritage, the integrated Computer Vision (CV) and Image Signal Processing (ISP) capabilities make it an especially appealing candidate for the star tracker. This is because the lower power visual processing for which the device was designed can be employed for the star tracker pre-processing stages, where star centroid information is extracted from a raw celestial scene capture. Through its dedicated ISP hardware blocks and parallel vector processors, the Myriad 2 can perform pre-processing followed by NN inference, providing an integrated star tracking compute solution in a monolithic processor.

The STM32 is based on an ARM Cortex M7 architecture that has seen extensive deployment in terrestrial embedded applications. STMicroelectronics provides a toolchain [10] that has made it a popular choice for lower-power 32-bit computing systems. To the authors’ knowledge, this device has not undergone any radiation characterization to date. However, it is a suitable low-cost comparative processor for the evaluation of the star tracker approach since it has an extensive toolchain for NN inference implementation at low power. The star tracker NN is at the lower end of the NN complexity spectrum, enabling the candidate networks to be ported for STM32 on-device inference.

Through the results of the power measurement experiment, conclusions have been drawn as to which processor is more efficient in terms of power and performance. These results contribute to choosing the best processor for building a low power star tracker. In this manuscript, a pattern feature extraction based star identification algorithm has been used which is discussed in detail in the following subsection.

### 2.2. The Star Identification Algorithm

The method of star tracking consists of three main steps i.e., centroiding, star identification, and attitude determination. Centroiding takes an image of the sky from the camera and determines the coordinates of the stars in that image. Star identification is one of the most crucial components of the star tracker [4]. The algorithm behind star identification takes the star centroid locations and magnitudes as inputs. The information is then compared to an internal star catalog to identify the present stars. The present stars are then passed onto an attitude determination algorithm. The lost-in-space star identification algorithms can identify the stars in a star scene without prior attitude information. These algorithms can be classified in terms of the feature extraction method choice. There are two of these main categories—pattern and subgraph isomorphism-based feature extraction [5]. In this manuscript, a pattern feature extraction based method as presented in [6], is used. The pattern feature extraction algorithms assign each reference star to a pattern based on the stars surrounding it and find the closest matching pattern in a database. Since neural network methods of star identification algorithms eliminate the number of searches required to match star patterns in the guide star database, these methods provide good analytical performance. The analytical performance of these methods is justified by the constant search time of O(1) [5].

The method used in this work makes use of a fully connected Neural Network to identify stars in a scene thereby returning the star labels. This particular network has been chosen since it can classify 2306 stars and can be implemented on existing high Technology Readiness Level (TRL) hardware, having TRL 9. Moreover, time expensive database searches required in the case of classical star identification algorithms are removed from the process since the star patterns are implicitly saved in the network. The algorithm also proves to be resilient to noise and false stars [6].

## 3. Experimental Setup

To verify the performance of the two processors over the pattern feature extraction star identification algorithm, several steps were performed. In this section, the processor specifications of the hardware and a detailed explanation of the chosen algorithm have been outlined along with the reason behind choosing them. The training and testing of the algorithm alongside power measurement experimental steps have also been discussed in the section. Since this manuscript is aimed to evaluate power measurement results on the Myriad 2 VPU and the STM32 Microcontroller on an NN-based star identification algorithm, deep learning development hardware was used in this experiment. The Intel Movidius Neural Compute Stick (NCS) consisting of the Myriad 2 VPU and the OpenMV Cam H7 Plus consisting of the STM32 Microcontroller was used.

### 3.1. Hardware Specifications

- Myriad 2 and the NCS: As highlighted in the Introduction, the Myriad 2 VPU is capable to withstand the space radiation environment. As a system-on-chip (SoC) family of devices, Myriad 2 [11] not only delivers enhanced computational performance and image processing functionality but also produces a low-power footprint. The Myriad 2 VPU is a high throughput multi-core architecture which is based on 12 Very Long Instruction Word (VLIW) 128-bit vector Streaming Hybrid Architecture Vector Engine (SHAVE) Cores that can run computations in parallel vector processors optimized for machine vision. It has configurable hardware accelerators for image and vision processing along with line buffers enabling zero local memory access in ISP mode. It contains 20 power islands containing low power states hence enabling us to perform low power computer vision and perform nominal 600 MHz operation at 0.9 V. Therefore, as mentioned in Section 2, through its dedicated ISP hardware blocks and parallel vector engines, the Myriad 2 can perform pre-processing followed by NN inference, providing an integrated star tracking system in a monolithic processor computing system.
  The NCS is based on the 4 GB Myriad 2 VPU. It is a miniature deep learning hardware development platform that can be used to prototype, tune, and validate AI programs, specifically deep neural networks. It provides high performance on low power devices for a very reasonable price. It is a real-time fanless inference device and helps perform inference on the edge rather than the cloud. It supports operating systems such as Windows 10, 64-bit*, Ubuntu 16.04*, and CentOS 7.4*. One of the reasons why the NCS has been chosen for the power measurement benchmark is that it does not require an additional power supply and it can deliver low cost and low power inference on deep learning algorithms.
- The STM32 Microcontroller and the OpenMV Cam H7 Plus: Under the STM32 Microcontroller [12], specifically, the STM32H743II ARM Cortex M7 processor was used. The STM32H743II contains STM32H742xI/G and STM32H743xI/G devices which are based on the high-performance Arm Cortex-M7 32-bit RISC core operating at up to 480 MHz. The Cortex -M7 core features a Floating Point Unit (FPU) which supports Arm double-precision (IEEE 754 compliant) and single-precision data-processing instructions and data types [12]. These devices support low-power consumption by use of VBAT battery operating mode with charging capability and CPU and domain power state monitoring pins.
  For this experiment, the OpenMV Cam H7 Plus is used as hardware which is based on the STM32H743II ARM Cortex M7 processor. It is a small, low power Microcontroller board through which real-world machine vision applications can be implemented. [13]. The OpenMV Cam can be programmed using high Python scripts making it easier to deal with complex outputs of the machine vision algorithms. The I/O pins on the OpenMV Cam are controllable in the Python scripts giving us a wide variety of functionality of using the hardware. The OpenMV Cam comes with a removable camera module making the OpenMV Cam H7 interface with different sensors. However, it should be noted that we are not making use of the image sensor on the OpenMV Cam to perform a fair comparison between the two processors chosen in this experiment. Please refer Table 1 for the detailed specifications of the devices used.

### 3.2. Implementation of the Algorithm

Before the star identification algorithm is implemented, the star centroiding algorithm is used in a star tracker which provides sub-pixel accuracy on the centroid determination of stars present in a scene. To pass the star centroids to the neural network, a feature extraction needs to be performed.

A simple pattern feature extraction method is used in this work as proposed in [6]. Specifically, a rotational-invariant pattern feature extraction is implemented since a neural network requires an input of invariant size and order. Many different approaches can be used to create such patterns. Due to the possibility of choosing the wrong reference star, the pattern is also very sensitive to noise. Hence, it is important to use the right approach. The method used in this work is similar to the Polestar algorithm [14] which uses the rotational invariant binned distances to the pattern stars from a centered polestar. This feature extraction is applicable to star identification using a neural network due to the fact that the binned distances are rotational invariant and the order of features is constant.

**Figure 1:**
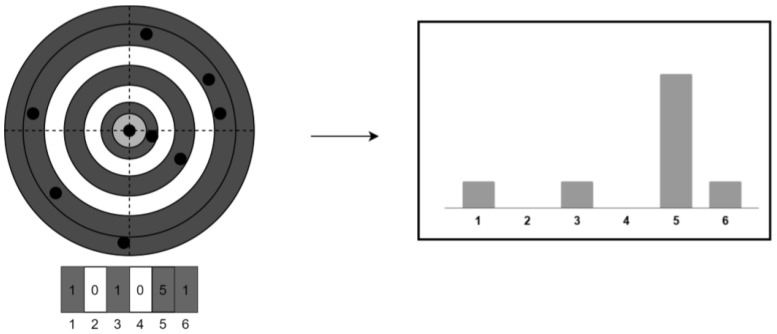
Feature extraction method used (reprinted from [6]).

In this work, a histogram of the binned distances was created to improve the performance of the neural network by reducing the variance. This histogram is then passed onto the network as proposed in [6] This approach delivers a low complexity (O(1)) yet efficient neural network that is also robust to false stars. The visual representation of the method is as shown in Figure 1.

**Figure 2:**
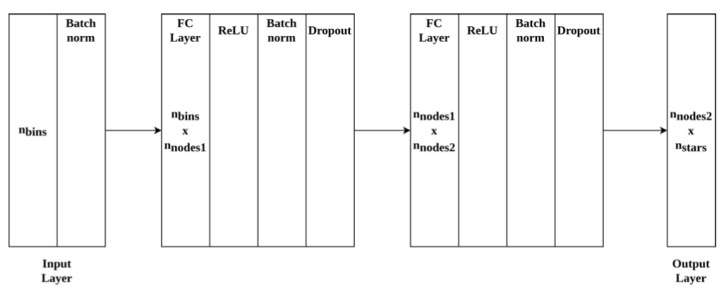
Neural network architecture used (reprinted from [6]).

The neural network architecture solves the tabular pattern recognition problem with the feature extraction method used [6]. The star identification problem has been approached as a classification problem and the network architecture is as shown in Figure 2 below. The network consists of an input layer depending on the number of bins followed by two fully connected layers and an output layer. Readers are encouraged to refer to the work [6] for further details on the network architecture.

### 3.3. Training

**Figure 3:**
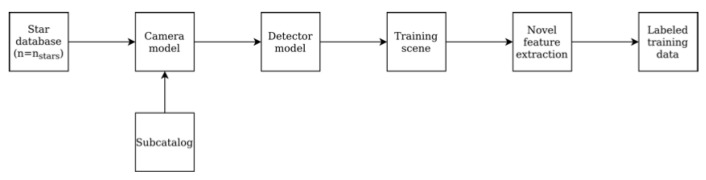
Training data generation method used (reprinted from [6]).

The networks were trained in Python and Keras framework using training data. The training data is generated similar to [6] by using a camera and a detector model the Hipparcos catalog [15], which is a star database made publicly available by the European Space Agency (ESA). A subsection of the star catalog is used and defined as the star database. Training data is generated by looping over the reference stars in this database. A scene consisting of an array of x, y coordinates of centroids is created using the camera and the detector model. This contains the reference star in the center. Relevant noise levels like the number of false stars per scene are also included. Table 2 shows the noise levels and training parameters used. Labeled data is then generated using the feature extraction method. The star ID number referring to the index of the reference star acts as the label. This labeled data then acts as input to the networks. Figure 3 shows the data generation process in detail.

To demonstrate that it is possible to use several different configurations to run them in different embedded devices, different sized networks were selected. The network sizes were specifically chosen considering the limited free space in the OpenMV hardware. The firmware that has to be included in the flash size of the device leaves 915 KB to store the network files. The size of the neural network was decided through the number of nodes in the network since this can directly influence the information it can store. The four network sizes used in the power measurement experiment include one network with 4096 nodes in both hidden layers, followed by one with 32 nodes in the first layer and 64 in the other, and the other two having 90 nodes per layer and 320 nodes per layer. The network sizes and descriptions have also been displayed in Table 2. A point to be noted here is that the classifier_320 was compiled in a ×4 factor compression format to fit in the free space of the flash memory of the OpenMV device.

It was also found that the classifer_4096 does not fit in the OpenMV Cam but it was run on the NCS to evaluate the results achieved. However, all of the networks can recognize 2306 stars from the catalog. This number of classes was chosen as it represents a brightness cut-off magnitude threshold of 5.3 Mv in the Hipparcos catalog.

### 3.4. Testing

After training the networks, certain steps were followed to perform the inference using the proposed hardware. The testing dataset used is similar to [6]. The parameters used in the testing set are outlined in Table 3. These also include the noise levels used to attain accuracy.

**Figure 4:**
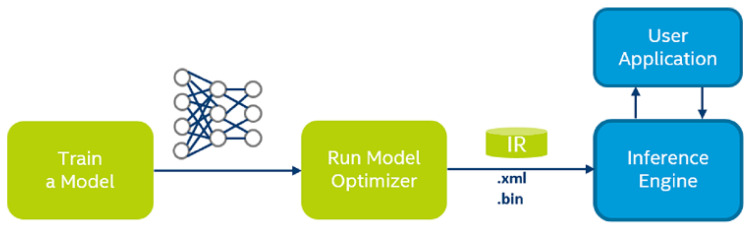
Converting networks to OpenVINO* IR format (reprinted from [16]).

To implement the networks using the NCS, the Intel OpenVINO Toolkit [16] was used. The trained networks were converted to TensorFlow format followed by their respective OpenVINO Intermediate Representation (IR) with the OpenVINO model optimizer as shown in Figure 4. The Model Optimizer returned the BIN and the XML files of the networks. The OpenVINO inference engine was then used to perform the inference using the NCS on the four networks.

**Figure 5:**
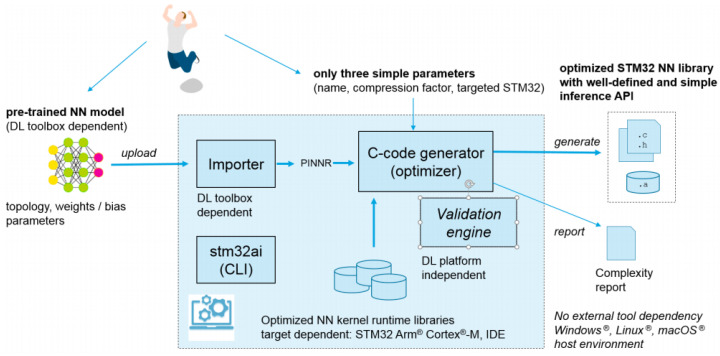
STMCubeMX* and CubeAI* workflow (reprinted from [17]).

**Figure 6:**
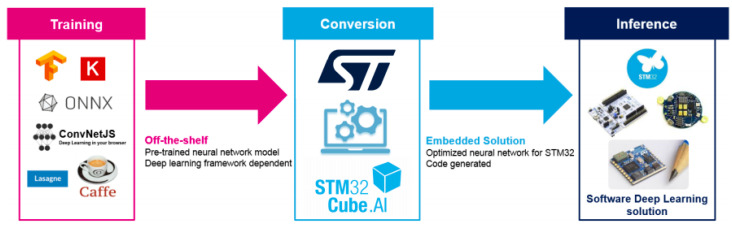
ST toolchain workflow (reprinted from [10]).

Secondly, to perform inference using the STM32 Toolchain on the OpenMV Cam, the Keras files were utilized in the STM32CubeMX [10] tool which converted them to C Code. This C-code generator optimizer also helps in optimizing memory usage for embedded devices against inference computation time and power consumption. The STMCubeMX is one of the applications of the STM32 Toolchain which creates an optimized neural network specifically for STM32 devices using the XCubeAI package. The workflow has been shown in Figure 5 and Figure 6. Four files were generated during this process for each network. Two of those files were data files for the weights/bias whereas the others were for the topology.

**Figure 7:**
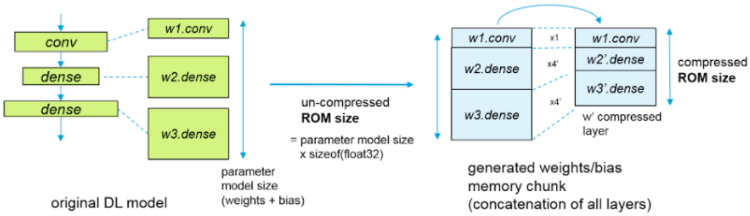
ST* compression (reprinted from [17]).

Figure 7 shows the network compression of the STM32CubeMX and CubeAI that performs three types of optimizations, the weight/bias compression, operation fusing, and optimal activation. The weight/bias compression is applicable for fully connected layers and uses the K-means clustering algorithm for quick data compression. The operation fusing merges the layers to optimize data placement. Some layers such as Reshape and Dropout are removed whereas other layers such as Nonlinearities, Conv, and Pooling are fused. In the optimal activation compression, a chunk is used to store temporarily hidden layer values. The RAM size is returned as the summation of the hidden activation size layers and the layer computing needs [17].

### 3.5. Power Measurement

After loading the networks for running the inference, the YZX Studio USB tester [18] along with the hardware devices were used to measure the power consumption for each processor.

In the case of the NCS, The YZX Studio USB Power tester was plugged along with the Intel NCS plugged onto it in one of the USB ports of the Intel NUC. To get accurate power measurement results, a Series Bluetooth Terminal Application [19] was used. This app was connected to the YZX Studio USB Power Tester via Bluetooth to our mobile device. This helped in getting access to the exact power measurement results along with the Voltage(V) and Current(I) used. For the OpenMV Cam, the network files were added to the OpenMV firmware project. The whole project was compiled and flashed onto the hardware. Then, to attain the power measurement results for the OpenMV Cam, similar steps as the NCS were followed using the YZX USB tester.

## 4. Results

In this section, the results obtained from the power measurement experiment on both devices are discussed. After the testing was performed, the power measurement results were visualized for both the NCS and the OpenMV Cam.

### 4.1. Power Measurement

The average power consumption for each of the networks—classifier_32_64, classifier_90, classifier_320, and classifier_4096—can be seen in the Table 4. A gradual increase in power usage and the number of inferences per watt can be seen as we move forward from the smallest network—classifier_32_64—to the largest network—classifier_4096. This is true for both the devices, except in the case of the classifier_90 in the case of the OpenMV Cam where there is a sudden drop in power. However, as seen in the Table, the NCS consumes less power than the OpenMV Cam when the testing is performed on the star identification algorithm. Moreover, since the largest network does not fit the OpenMV memory requirements, results are not displayed for that particular network. As discussed earlier in Section 3, the classifier_320 had to be compressed by four times to fit the OpenMV memory requirements.

### 4.2. Accuracy vs. Power vs. Speed

**Figure 8:**
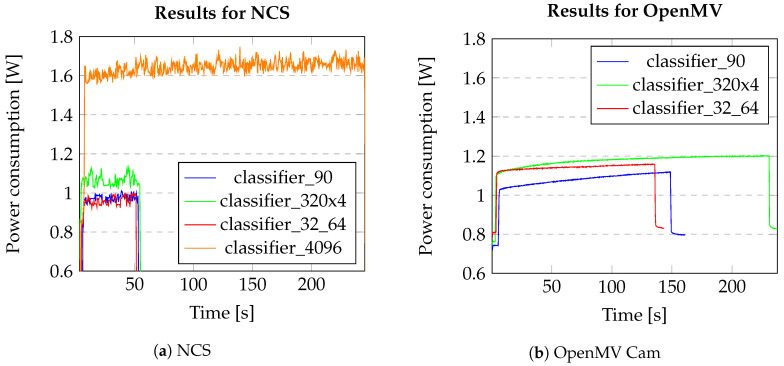
Power consumption vs. time taken for all the networks for 11,535 consecutive inferences.

Figure 8a,b show the power consumption for all the networks with respect to time in seconds, on both the devices. This shows the comparison of power consumption for 11,535 inferences for each network, thereby also giving an indication of their execution time. It can be seen from Figure 8a that, for the NCS, the largest network takes the most time and consumes the most power, while the remaining three networks have a shorter execution time and consume much less power. This is principally due to the size of the network—the increase in the number of nodes in the network leads to an increase in the power consumed to complete the multiply-accumulate and data transfer operations performed as part of inference. In the case of the OpenMV Cam, it can be seen that the classifier_320 takes the most time to execute the inferences, but that its peak power draw is not considerably different to that of the classifier_32_64 inferences.

The Table 5 shows how fast each of the networks performs on the devices in the form of FPS and also how accurate the networks were when run on these devices. The accuracy of the networks is the ratio of correct matches to total matches, expressed as a percentage. For both the NCS and the OpenMV Cam, there is a strict decrease in the FPS as the network size increases. In terms of accuracy there is a subtle increase in accuracy moving from the classifier_32_64 to the classifier_90 to the classifier_320 for both the devices.

There is a slight difference in accuracy between the models because of the network size and how it is optimized for the underlying hardware. Also, it is interesting to note that the accuracy is affected by the device used and is slightly different for both the devices. This is most likely due to quantization and optimization steps performed during the conversion and compilation processes necessary to run the networks on each specific hardware. Note that the accuracy of the networks was calculated with noise levels of 0–4 stars per scene.

**Figure 9:**
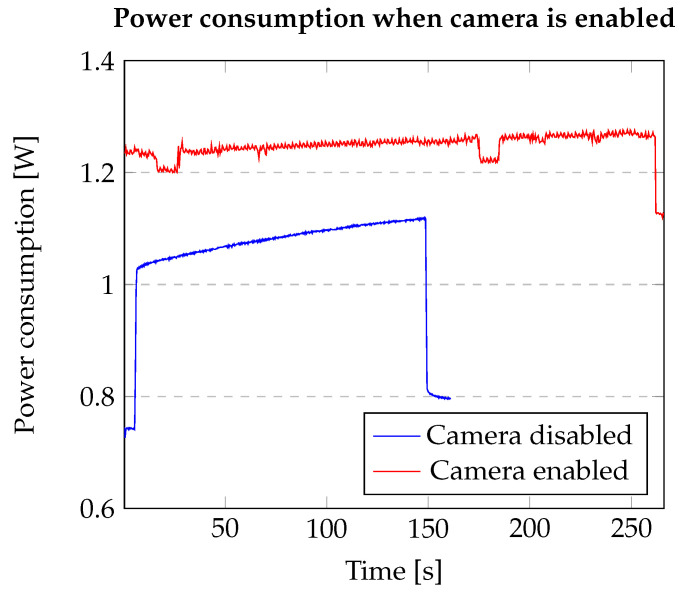
Results for the experiment with the camera activated on the OpenMV Cam.

It should also be noted that the OpenMV Cam device containing the SMT32 Microcontroller has several sensors including the OV5640 image sensor. In this work, the image sensors and the camera were not used so that results could be evaluated similarly for both the processors without additional limitations. Figure 9 shows that the power consumption when the camera is enabled would be much higher and in this case, the camera has not been used.

### 4.3. Robustness of the Algorithm Used

Another point to be highlighted here is the robustness of the star identification algorithm used in this work. Star sensor systems can be exposed to different types of noise. As mentioned above, positional noise and false stars are considered in this work. To be robust, the star identification algorithm needs to be able to deal with such noise levels. The robustness of the algorithm has been tested in [6] by increasing the amount of false star percentages in each step. The networks—classifier_32_64 and the classifier_4096—were tested in a false star experiment to determine which network performs better in a given condition. These were trained on 2306 star classes as mentioned in the Section 3. It was observed that the difference between both networks is negligible. However, in extreme application environments when up to 10% false stars are added, the larger network (classifier_4096) performs better than the smaller network (classifier_36_64).

## 5. Discussion

In this work, FPS, accuracy, and power consumption have been used as benchmarks to evaluate the performance and efficiency of two COTS processors for the NN-based start tracker task.

As seen in Table 5, there is a gradual increase in accuracy going from the smallest network—classifier_32_64 to the second-largest network—classifier_320. However, interestingly, for the largest network—classifier_4096—there is a slight unexpected drop in the accuracy of the network as well as the number of inferences per watt as shown in Table 4. This is only seen in the case of the Myriad 2, since the largest network does not fit within the OpenMV memory requirements. This accuracy drop may indicate that the training data size was insufficient to achieve stable values for the much larger number of weights in this network relative to the smaller networks. Furthermore, the accuracy of the networks on the two processors also shows a slight difference between them depending on the network, with a maximum absolute accuracy difference of 0.1% between devices. This accuracy difference is likely due to the toolchain optimisation steps performed when compiling the networks for the two devices. It is interesting to note that even though compression was used on classifier_320 when deployed on OpenMV, as shown in the Table 2 the effect on accuracy of this compression was not significant relative to the NCS accuracy.

The power consumption presented in Table 4 also shows a gradual increase going from the smallest network to the largest network, with the Myriad 2 having the lower power consumption results. However, there is a slight unexpected drop in power for the classifier_90 in the case of the OpenMV Cam. The FPS on the other hand decreases as we move from the smallest to the largest network. Regardless of the network, these results indicate that the Myriad 2 is more efficient than the STM32 at performing inference across the test networks. This is an important factor to consider when selecting a processing platform on which to implement the star tracker solution for SmallSats. The operating frequency of the star tracker (the rate at which it provides attitude updates) is dependent on the FPS of the implemented network, and ’fast’ star trackers would be better suited to implementation on the Myriad 2.

When it comes to striking a balance between accuracy, power, and the speed execution of the network, from Table 4 and Table 5, the classifier_320 with 99.08% accuracy, average power consumption of 1.06 ± 0.02 W and FPS of 224.09 when run on the Myriad 2 VPU may be the best choice for a low power star tracker. However, ultimately the best combination of network and processor is dependent on the overall star tracker system requirements (peak and average power, required FPS, and accuracy). If a trade-off can be made concerning the accuracy, then the classifier_32_64 exhibits the lowest power consumption. Nevertheless, the results in Section 4 show that the Myriad 2 VPU has consistently higher frame rate and lower power consumption than the STM32 across all evaluated networks.

## 6. Conclusions

In this manuscript, two cost-effective processors—the Myriad 2 VPU and the STM32 ARM Cortex M7 Microcontroller—were examined through a series of experiments. These experiments were carried out to evaluate each processor as a low-cost, low-power NN-based star tracker. Both processors demonstrated their capability to run star tracking neural networks, with the Myriad 2 achieving higher speed while consuming less power during operation.

One counter-intuitive result was that the accuracy dropped for the larger network—classifier_4096. This means that although the STM32 has more limited memory compared to the Myriad 2, it still achieves comparable maximum accuracy to the Myriad 2 (99.07% vs. 99.08%), indicating that, for the star tracker networks evaluated, the memory limitations of the STM32 are not a limiting factor—the larger memory size of the Myriad gave no additional advantage in accuracy for the large network size tested. While the Myriad 2 VPU was more power efficient at executing networks (0.89–1.08 W), the STM32 was certainly comparable in power usage (1.15–1.2 W). Although the Myriad 2 was capable of a higher frame rate, the STM32 still remains a viable contender for low-cost star trackers.

The final caveat is that the STM32 Microcontroller, unlike the Myriad 2, has not undergone any radiation testing to-date, per the authors’ knowledge. Nonetheless, the results of this evaluation have shown that it is a suitable low-cost comparative processor for the implementation of an NN-based star tracker. Firmware available for the OpenMV Cam indicates that, like the Myriad 2, the STM32 is adept at executing classical Computer Vision (CV) pipelines that may facilitate the execution of the image processing required for the front-end of the star tracker, albeit that it does not have the Myriad 2’s CV hardware acceleration features. Accordingly, the STM32 may be considered a candidate for radiation characterisation for use in future star tracker space applications. The SPC58 MCU automotive family from STMicroelectronics may also warrant evaluation for the star tracker task as this family has been radiation tested.

As mentioned in Section 2, the positive radiation characterization results of the Myriad 2 VPU, and the hardware-accelerated and parallelised CV and ISP capabilities, make it suitable for the practical implementation of the complete SmallSat star tracker processing chain. The STM32 is a more general general processor than the Myriad 2, but, with the use of the XCubeAI package, it has been shown to be applicable to the NN-based star tracker task. Hence, this work not only highlights capable, low-cost, low-power COTS star tracker solutions, but also a methodology for evaluating star tracker performance on suitable hardware.

Future work would examine the use of different networks for star trackers, particularly in the context of the identified accuracy loss that was seen for the larger network. Construction and validation of a full end-to-end star tracker pipeline, including full feature extraction, along with evaluation of other processors for their suitability in executing the star tracker task, are additional future work topics.

## 7. Patents: Intel Legal Disclaimers, Notices and Disclosures

Software and workloads used in performance tests may have been optimized for performance only on Intel microprocessors. Performance results are based on testing as of dates shown in configurations and may not reflect all publicly available updates.

## Figures and Tables

**Table 1:** Hardware specification.

Device	NCS	OpenMV Cam H7 Plus
Processor	Intel Movidius Myriad 2 VPU	STM32H743II ARM Cortex M7
	(12 WLIW 128-bit vector SHAVE Cores)	32-bit RISC core
On-chip memory	4GB LPDDR3 SDRAM	32MBs SDRAM + 1 MB of SRAM
		and 32 MB external quadspi flash
		+ 2 MB of internal flash
Processor base frequency	933 MHz	480 MHz
Native support precission	FP16	FP64
External modules	None included	Removable OV5640 image sensor

**Table 2:** Training dataset parameters.

Parameter	Value
Number of classes	2306
Number of scenes per class	360
Number of false stars per scene	0–4 (randomly added)
Number of bins	25
Resolution of detector	1000 × 1000 pixels
Field of view (FOV) of camera	20 × 20 degrees
Cut-off magnitude threshold	5.3 $M_{\upsilon }$
Standard deviation of added Gaussian vector noise	$20\times 10^{-6}$
Standard deviation of added Gaussian magnitude noise	0.01 $M_{\upsilon }$
Standard deviation of added Gaussian positional noise	0 pixels

**Table 3:** Testing dataset parameters.

Parameter	Value
Number of classes	2306
Number of scenes per class	5
Number of false stars per scene	0–4 (randomly added)
Number of bins	25
Resolution of detector	1000 × 1000 pixels
Field of view (FOV) of camera	20 × 20 degrees
Cut-off magnitude threshold	5.3 $M_{\upsilon }$
Standard deviation of added Gaussian vector noise	$20\times 10^{-6}$
Standard deviation of added Gaussian magnitude noise	0.01 $M_{\upsilon }$
Standard deviation of added Gaussian positional noise	0 pixels

**Table 4:** Average power consumption and inference per watt results for each of the networks.

Star Tracker Model	Average Power Consumption (W)		Inference per Watt	
	NCS	OpenMV	NCS	OpenMV
classifier_32_64	0.98	1.15	268.80	104.92
classifier_90	0.99	1.10	234.11	98.89
classifier_320	1.08	1.20	210.53	50.84
classifier_4096	1.68	-	29.45	-

**Table 5:** Frames Per Second (FPS) vs. Accuracy results for each of the networks as expressed in percentage.

FPS Results				
Star Tracker Network	NCS		OpenMV	
	FPS	Accuracy	FPS	Accuracy
classifier_32_64	257	97.98	119.35	97.97
classifier_90	227.94	98.84	106.93	98.94
classifier_320	224.09	99.08	59.92	99.07
classifier_4096	48.48	98.96	-	-
